# Characteristics of pathogenic microorganisms in COPD-related infections: prognostic correlations and implications

**DOI:** 10.3389/fcimb.2025.1739849

**Published:** 2026-01-19

**Authors:** Chaoying Liu, Caihong Liu, Huibo Liu, Shan Lin

**Affiliations:** 1 Department of Respiratory Medicine, The First Hospital of Jilin University, Changchun, China; 2 Department of Laboratory Medicine, The First Hospital of Jilin University, Changchun, China; 3 Department of Dermatology, The First Hospital of Jilin University, Changchun, China

**Keywords:** COPD, infection, machine learning, next-generation sequencing, pathogenic microorganism, prognosis

## Abstract

**Background:**

Chronic obstructive pulmonary disease (COPD) significantly impacts global health, primarily due to frequent acute exacerbations caused by respiratory infections. Precise microbial characterization may inform prognostic insights and optimize clinical management.

**Methods:**

We conducted a prospective observational study from December 2023 to February 2025 involving 1146 patients (259 COPD; 887 non-COPD) with suspected respiratory infections. Bronchoalveolar lavage fluid samples underwent next-generation sequencing (NGS) and conventional microbiological testing. Multivariate logistic regression identified COPD predictors, and machine learning modeled prognostic outcomes based on microbial profiles.

**Results:**

Distinct pathogen distributions emerged between COPD and non-COPD groups, with COPD patients exhibiting higher prevalence of gram-negative bacteria, particularly *Pseudomonas aeruginosa* and *Haemophilus influenzae*, and fungal pathogens. Non-COPD patients demonstrated increased occurrence of atypical pathogens, notably *Mycoplasma pneumoniae*. COPD patients also presented higher loads of traditionally commensal microorganisms, such as *Veillonella parvula* and *Schaalia odontolytica*. Age, dyspnea, smoking duration, elevated leukocyte and neutrophil counts, and decreased lymphocyte levels were significantly associated with COPD presence. Machine learning identified specific microorganisms as strong predictors of adverse outcomes, such as SARS-CoV-2, *Veillonella parvula*, and *Achromobacter xylosoxidans*.

**Conclusions:**

Comprehensive microbial profiling using NGS effectively distinguishes pathogen differences between COPD and non-COPD patients, revealing key associations with clinical prognosis. These insights can inform tailored clinical interventions aimed at mitigating COPD exacerbations and improving patient outcomes.

## 1 Introduction

Chronic obstructive pulmonary disease (COPD) represents a major global health burden, characterized by persistent respiratory symptoms and progressive airflow limitation, resulting from chronic inflammation of the airways and lung parenchyma (Bigna et al., 2017; Wang et al., 2022). As the third leading cause of death worldwide, COPD significantly impairs patients’ quality of life, incurs substantial healthcare expenditures, and imposes a considerable socioeconomic burden (Lytsy et al., 2022; Florman et al., 2023). Among COPD-related complications, acute exacerbations are particularly critical, contributing substantially to disease progression, increased hospitalization rates, accelerated lung function decline, and heightened mortality (Puhan et al., 2016; Jain et al., 2023). Respiratory infections are established as predominant precipitants of acute exacerbations in COPD, with bacterial, viral, and atypical pathogens frequently implicated (Tian et al., 2018; Linden et al., 2019; Liao et al., 2022; Sonowal and Shariff, 2024). However, the spectrum and distribution of infectious microorganisms among COPD patients compared with non-COPD individuals, as well as their potential prognostic significance, remain inadequately elucidated.

Recent technological advancements in microbial identification, particularly next-generation sequencing (NGS), offer unprecedented sensitivity and specificity in the detection and characterization of microorganisms (Fourgeaud et al., 2023; Yin et al., 2024). Compared to traditional culture-based methods, NGS provides comprehensive microbiome profiles, enabling more accurate identification of microorganisms, thereby deepening our understanding of the microbial landscape in COPD patients, informing targeted antimicrobial therapies, and potentially improving clinical management strategies aimed at reducing exacerbation frequency and severity.

Therefore, this study aims to systematically investigate the differences in infectious pathogen profiles between COPD and non-COPD patients, and further explore the associations between pathogen-specific infections and adverse clinical outcomes in COPD patients. Our findings may provide critical insights into disease mechanisms underlying COPD exacerbations and facilitate the development of precise clinical interventions aimed at optimizing patient prognosis.

## 2 Methods

### 2.1 Study design and participants

This study was conducted at the Department of Respiratory Medicine, the First Hospital of Jilin University. Patients with suspected respiratory infection who were admitted to our department between December 2023 and February 2025 were consecutively included. Inclusion criteria encompassed (1) suspected lower respiratory infection (defined as a new-onset radiological findings on chest images or a hematologic parameter abnormality combined with at least one compatible symptom, such as fever, cough, or dyspnea); (2) need for bronchoalveolar lavage according to clinical standard procedure; (3) sufficient bronchoalveolar lavage fluid (BALF) samples for the NGS and conventional microbiological tests (CMTs, including BALF culture, PCR, and serological tests). Patients with insufficient clinical information or inavailable BALF sample were excluded. Enrolled patients were divided into two groups based on whether they had COPD status or not. The diagnosis of COPD status was according to the Global Initiative for Chronic Obstructive Lung Disease (GOLD) guidelines (GOLD, 2025). Fluid from the middle segment of bronchoalveolar lavage was collected as the study sample. All BALF samples underwent conventional microbiological testing, and remaining aliquots were preserved for NGS.

This study design received approval from the Ethics Committee of the First Hospital of Jilin University in accordance with the Declaration of Helsinki (2025-396). The study was conducted with the consent of every human participant.

### 2.2 Clinical data collection

Detailed demographic and clinical data were extracted from electronic medical records, including age, gender, immune status (immunocompetent, immunocompromised by cancer chemotherapy, and immunocompromised by diabetes), dyspnea, smoking history, hematologic parameters (white blood cell [WBC], neutrophil [NE], lymphocyte [LYM], C-reactive protein [CRP], and procalcitonin [PCT]), and prognosis parameters (days of hospitalization, intensive care unit [ICU] admission, mechanical ventilation, and outcome [survival and death]). Poor prognosis was defined as the presence of any of ICU admission, mechanical ventilation, and death.

### 2.3 Next-generation sequencing

Before extracting the nucleic acid, host DNA was first depleted from BALF samples using the MolYsis™ Basic 5 Kit (Molzym GmbH & Co. KG, Bremen, Germany). Nucleic acids were then extracted using the Magnetic Pathogen DNA/RNA Kit (Tiangen Biotech [Beijing] Co., Ltd, Beijing, China). Following this, the RNA was reverse-transcribed into cDNA using the Hieff NGS Double Stranded cDNA Synthesis Kit (Yeasen, Shanghai, China). The concentration of the total DNA was quantified using the Qubit™ Double-Stranded DNA High Sensitivity Assay Kit (Thermo Fisher Scientific Inc., Waltham, MA, USA). Then DNA libraries were prepared using the VAHTS Universal Plus DNA Library Prep Kit for MGI (Vazyme, Nanjing, China) with an initial input of 2 ng. Meanwhile, library quality control was performed with the Agilent 2100 Bioanalyzer (Agilent Technologies Inc., Santa Clara, CA, USA) to evaluate DNA concentration and fragment size distribution. Libraries exhibiting a dominant fragment peak between 240 bp and 350 bp and a concentration exceeding 1 ng/μL were deemed qualified. Approved libraries were pooled, denatured, and circularized to form single-stranded DNA circles. DNA nanoballs (DNBs) were subsequently generated through rolling circle amplification. The resulting DNBs were loaded onto sequencing chips and subjected to single-end 50-bp sequencing on the BGISEQ-500 (BGI, Shenzhen, China) platform, yielding approximately 10 to 20 million reads per library.

Following sequencing, low-quality reads, short reads, and adapter were removed using Fastp (version 0.23.4) to generate high-quality data for downstream analysis. Clean reads were aligned to three human reference genomes (hg38, T2T-CHM13, and YH1) using Burrows-Wheeler Aligner (BWA, version 0.7.17-r1188). Human-derived reads were subsequently excluded using Samtools (version 1.6). Then the remaining reads were aligned against a custom microbial database using BWA to derive annotations (**Supplementary Table S1**). After that, the annotation results were further validated by BLAST (version 2.12.0) to ensure accuracy.

### 2.4 Clinical diagnosis

Microorganisms identified by NGS were independently evaluated by a minimum of three clinicians holding at least an associate senior professional title, and classified into four levels, including causative pathogen, possibly causative pathogen, microorganism without pathogenic role, and not causative pathogen (Fourgeaud et al., 2023). The clinical diagnosis was established by clinicians following a comprehensive assessment that integrated patient age, gender, immune status, medical history, symptoms, hematologic parameters, radiological findings, CMT results, prognosis, and other relevant clinical data (**Supplementary Figure S1**).

### 2.5 Statistical analysis

For descriptive statistics, continuous variables were presented as medians and interquartile ranges (IQR), whereas categorical variables were reported as frequencies and percentages. Multivariate logistic regression was used to compute the odds ratio (OR) and 95% confidence interval (CI) for the associations of gender, age, immune status, symptoms, smoking history, hematologic and prognosis parameters with the presence of COPD. Data normality was assessed by the Shapiro-Wilk test. Nonparametric variables were compared using Mann-Whitney test. All tests were two-tailed and significance threshold was set at p value ≤ 0.05. All statistical analyses were performed using SPSS Statistics (version 26.0, IBM Corp., Armonk, NY, USA). All figures were drawn using R software (version 4.3.1, R Foundation for Statistical Computing, Vienna, Austria), Python (version 3.11, Python Software Foundation, Wilmington, DE, USA), and GraphPad Prism (version 9.5.0, GraphPad Software LLC., San Diego, CA, USA).

## 3 Results

### 3.1 Study participants

**Figure 1:**
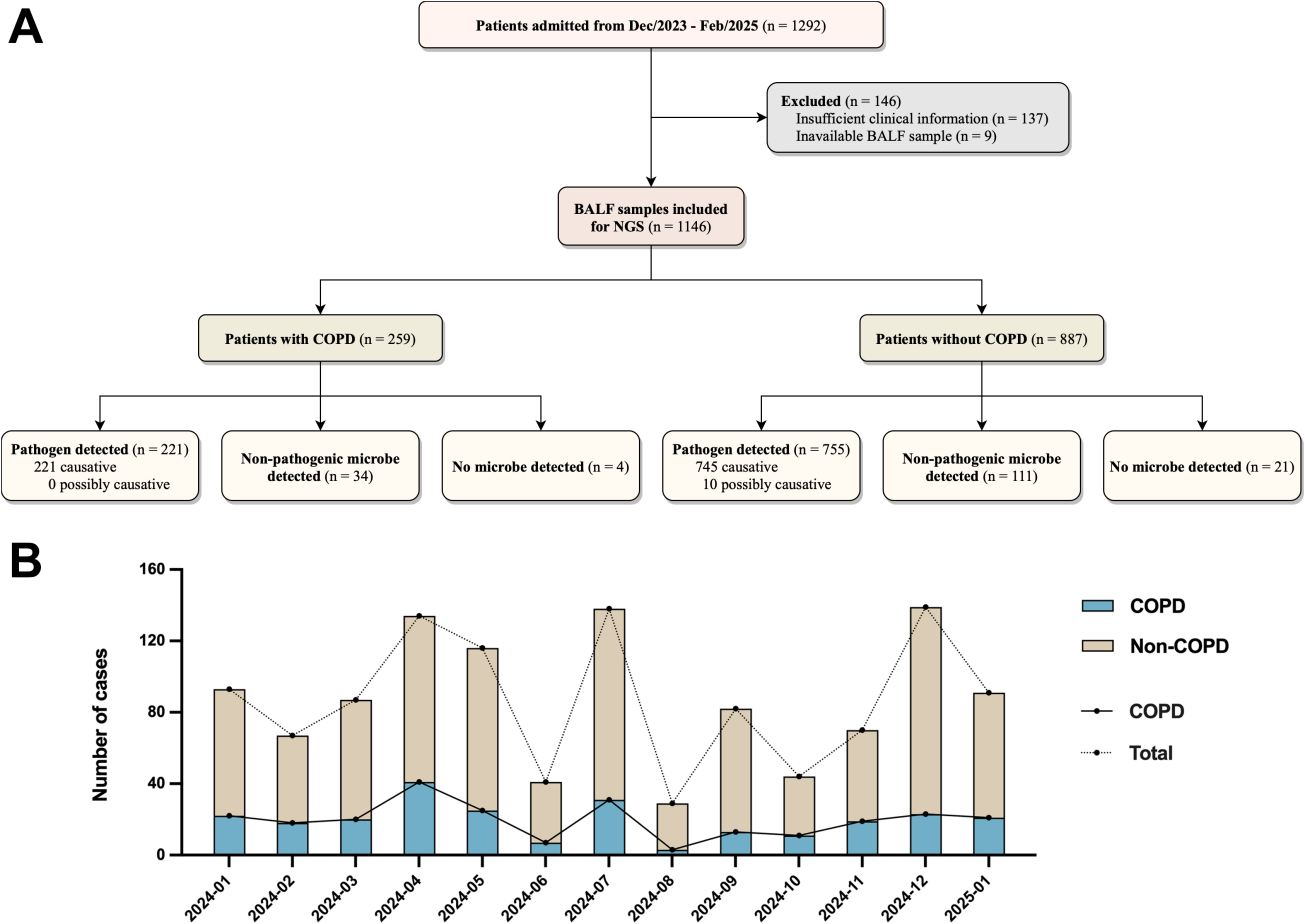
Patients included in this study. **(A)** Flowchart of patients included and excluded. BALF, bronchoalveolar lavage fluid; NGS, next-generation sequencing; COPD, chronic obstructive pulmonary disease. **(B)** Patients with respiratory infections enrolled between January 2024 and January 2025 and the percentage of COPD patients among them.

In this study, a total of 1292 patients with suspected respiratory infection were screened between December 2023 and February 2025 (**Figure 1A**). Among this, 146 cases were excluded due to insufficient clinical information (n = 137) and inability to collect BALF samples (n = 9). BALF samples of 1146 patients were analyzed using NGS. In patients with COPD (22.6%, 259/1146), NGS detected causative pathogens in 221 samples, microorganisms without pathogenic role in 34 samples, and no microorganisms in 4 samples. Whereas in patients without COPD (77.4%, 887/1146), NGS detected causative pathogens in 745 samples, possibly causative pathogens in 10 samples, microorganisms without pathogenic role in 111 samples, and no microorganisms in 21 samples (**Supplementary Table S2**). The monthly incidence of respiratory infection episodes (median 87 [IQR 67 - 116] cases) remained relatively consistent throughout the study period, with modest increases observed in April (n = 134), May (n = 116), July (n = 138), and December (n = 139) (**Figure 1B**). The proportion of cases involving patients with COPD among those presenting with respiratory infections also demonstrated temporal stability, with a median prevalence of 22.99% (IQR 17.07% - 25.00%).

The demographic and clinical characteristics of patients in this study were shown in **Table 1**. The median age of the overall cohort was 66 (IQR 55 - 73) years, with patients in COPD group being older (median 70 [IQR 66 - 78] years) compared to those without COPD (median 63 [IQR 52 - 71] years; p < 0.0001). Regarding gender distribution, females accounted for 44.1% (505/1146) of the total population, with a slightly higher proportion in COPD group (48.3%, 125/259) compared to non-COPD group (42.8%, 380/887; p > 0.05). In terms of immune status, the majority of patients were immunocompetent (77.9%, 893/1146), with comparable rates between COPD (81.9%, 212/259) and non-COPD (76.8%, 681/887) groups (p > 0.05). The immunocompromised cohort primarily comprised patients with diabetes mellitus (16.5%, 189/1146) and patients undergoing chemotherapy for cancer (5.6%, 64/1146). Among patients with COPD, the proportion of smoking was higher (49.4% *vs*. 32.8%; p < 0.0001), and the duration of smoking history was longer (median 10 [IQR 0 - 37.5] *vs*. 0 [IQR 0 - 20] years; p < 0.0001), compared to that in patients without COPD. For hematologic parameters, the WBC count (median 10.3 [IQR 7.3 - 13.7] *vs*. 8.9 [IQR 5.4 - 13.0] 10^9^/L; p = 0.0068) and NE percentage (median 83.2% [IQR 71.6% - 90.3%] *vs*. 78.8% [IQR 62.5% - 88.6%]; p < 0.0001) were both higher in COPD group than in non-COPD group. The median CRP and PCT were 45.8 (IQR 9.7 - 110.0) mg/L and 0.116 (IQR 0.057 - 0.412) ng/mL, respectively, with comparable values between COPD (median 44.5 [IQR 9.6 - 121.9] mg/L for CRP; median 0.108 [IQR 0.054 - 0.349] ng/mL for PCT) and non-COPD (median 46.3 [IQR 9.9 - 107.7] mg/L for CRP; median 0.117 [IQR 0.058 - 0.425] ng/mL for PCT) groups (p > 0.05). With respect to duration of hospitalization and mortality, no significant differences were observed between COPD and non-COPD groups (p > 0.05). In the overall cohort, the median length of hospital stay was 8 (IQR 6 - 11) days, and the mortality rate was 4.8% (55/1146). However, the ICU occupancy (15.44% *vs*. 10.94%, p = 0.04901) and mechanical ventilation utilization (12.36% *vs*. 4.4%, p < 0.0001) of COPD group were significantly higher than that of non-COPD group.

**Table 1:** Demographic and clinical characteristics of patients across COPD and Non-COPD groups.

Index	Overall (n = 1146)	COPD (n = 259)	Non-COPD (n = 887)	P value
Age, median (IQR) (years)	66 (55 - 73)	70 (66 - 78)	63 (52 - 71)	< 0.0001
Gender				
Female, n (%)	505 (44.1%)	125 (48.3%)	380 (42.8%)	0.1221
Male, n (%)	641 (55.9%)	134 (51.7%)	507 (57.2%)	0.1221
Immune status				
Immunocompetent, n (%)	893 (77.9%)	212 (81.9%)	681 (76.8%)	0.0830
Cancer, n (%)	64 (5.6%)	12 (4.6%)	52 (5.9%)	0.4485
Diabetes, n (%)	189 (16.5%)	35 (13.5%)	154 (17.4%)	0.1420
Dyspnea, n (%)	411 (35.9%)	198 (76.4%)	213 (24.0%)	< 0.0001
Smoking history				
Smoking, n (%)	419 (36.6%)	128 (49.4%)	291 (32.8%)	< 0.0001
Smoking, median (IQR) (years)	0 (0 - 30)	10 (0 - 37.5)	0 (0 - 20)	< 0.0001
Hematologic parameters				
WBC, median (IQR) (10^9/L)	9.2 (5.5 - 13.2)	10.3 (7.3 - 13.7)	8.9 (5.4 - 13.0)	0.0068
NE, median (IQR) (%)	79.8 (65.1 - 89.1)	83.2 (71.6 - 90.3)	78.8 (62.5 - 88.6)	< 0.0001
LYM, median (IQR) (%)	12.0 (6.0 - 20.9)	9.7 (5.3 - 16.3)	12.8 (6.4 - 22.6)	< 0.0001
CRP, median (IQR) (mg/L)	45.8 (9.7 - 110.0)	44.5 (9.6 - 121.9)	46.3 (9.9 - 107.7)	0.3306
PCT, median (IQR) (ng/mL)	0.116 (0.057 - 0.412)	0.108 (0.054 - 0.349)	0.117 (0.058 - 0.425)	0.9278
Prognosis				
After admission, median (IQR) (days)	8 (6 - 11)	8 (6 - 10)	8 (6 - 11)	0.2008
ICU, n (%)	137 (11.95%)	40 (15.44%)	97 (10.94%)	0.0491
Mechanical ventilation, n (%)	71 (6.2%)	32 (12.36%)	39 (4.4%)	< 0.0001
Dead, n (%)	55 (4.8%)	9 (3.5%)	46 (5.2%)	0.2570

COPD, chronic obstructive pulmonary disease; IQR, interquartile range; WBC, white blood cell; NE, neutrophil; LYM, lymphocyte; CRP, C-reactive protein; PCT, procalcitonin; ICU, intensive care unit.

### 3.2 Significant correlation between COPD and poor prognosis

**Figure 2:**
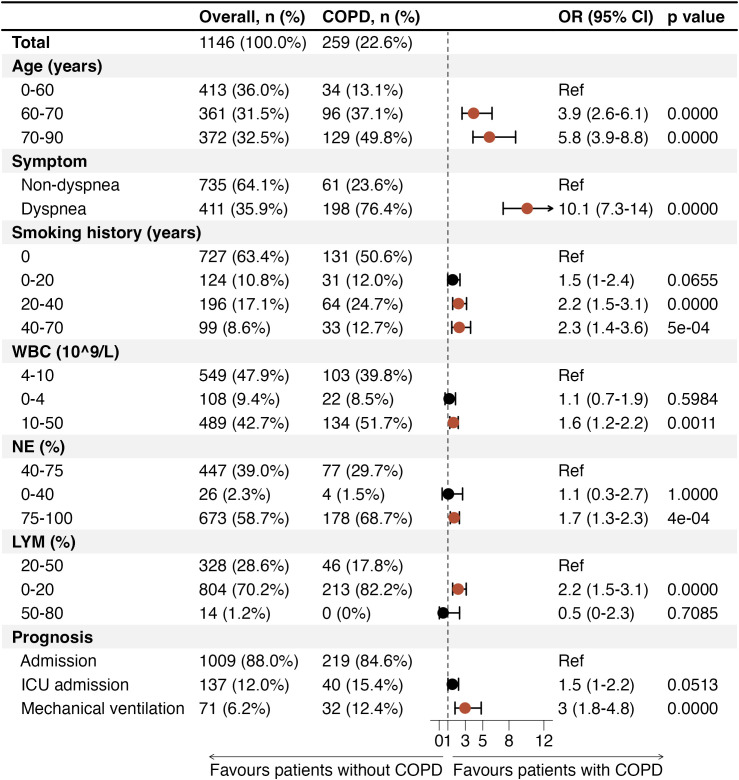
Multivariate regression analysis of COPD and clinical parameters. Only p values less than 0.05 were considered significant and are indicated by red dots. COPD, chronic obstructive pulmonary disease; OR, odds ratio; CI, confidence interval; Ref, reference; WBC, white blood cell; NE, neutrophil; LYM, lymphocyte; ICU, intensive care unit.

Multivariate logistic regression analysis was performed to clarify the associations of demographic and clinical characteristics of patients with the presence of COPD among those with respiratory infections (**Figure 2**). Age was a strong predictor: compared with patients younger than 60 years, those aged 60–70 years had nearly a four-fold higher odds of COPD (OR 3.9, 95% CI 2.6 - 6.1, p value < 0.0001), and those aged 70–90 years had almost six-times the odds (OR 5.8, 95% CI 3.9 - 8.8, p value < 0.0001). Dyspnea was significantly associated with COPD (OR 3.8, 95% CI 2.6 - 5.5, p value < 0.0001). A longer history of smoking was also independently associated: compared with never-smokers, those with 20–40 years of smoking had more than double the odds (OR 2.2, 95% CI 1.5 - 3.1, p value < 0.0001), and those with 40–70 years had a similar increase (OR 2.3, 95% CI 1.4 - 3.6, p value = 0.0005). Laboratory markers and prognosis parameters differed significantly in COPD patients with respiratory infections (**Figure 2**). These patients demonstrated significantly higher odds of elevated WBC counts (10 - 50 10^9^/L, OR 1.6, 95% CI 1.2 - 2.2, p value = 0.0011), as well as increased NE (75% - 100%, OR 1.7, 95% CI 1.3 - 2.3, p value = 0.0004) and decreased LYM (0% - 20%, OR 2.2, 95% CI 1.5 - 3.1, p value < 0.0001) levels. The correlation between ICU admission and COPD approaches significance (p = 0.0513). Notably, COPD patients were three times more likely to require mechanical ventilation (OR 3, 95% CI 1.8 - 4.8, p value < 0.0001).

### 3.3 Characterization of causative pathogens

**Figure 3:**
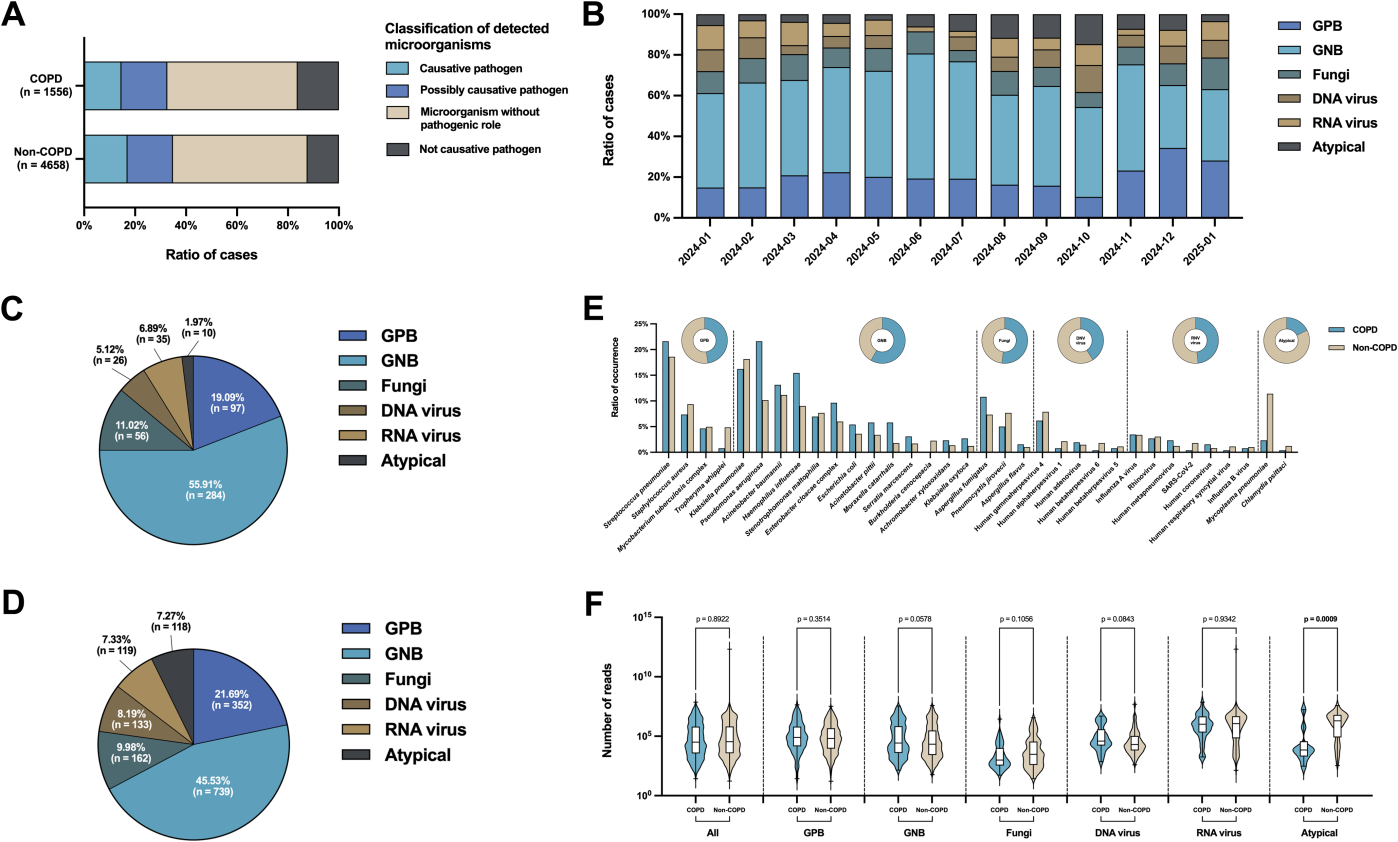
Distribution of microorganisms. **(A)** All microorganisms identified by NGS in patients with and without COPD, along with the proportional distribution across each corresponding level of clinical diagnosis. **(B)** Proportional distribution of each species type of causative or possibly causative pathogens detected between January 2024 and January 2025. GPB, gram-positive bacteria; GNB, gram-negative bacteria. **(C, D)** Proportional distribution of each species type of causative or possibly causative pathogens detected in patients with and without COPD. **(E)** Occurrence of each species of causative or possibly causative pathogens detected in patients with and without COPD. Circles represent the occurrence of each species type of causative or possibly causative pathogens detected in patients with and without COPD. **(F)** Distribution of reads counts of causative or possibly causative pathogens detected by NGS in COPD versus non-COPD groups, overall and across each species type.

Based on the complete spectrum of microorganisms identified by NGS, the distribution of clinical diagnostic classifications was broadly comparable between patients with and without COPD (**Figure 3A**). In both cohorts, “microorganisms without pathogenic role” comprised the single largest category (51.22% for COPD; 52.90% for non-COPD), followed by “possibly causative pathogens” (17.99% for COPD; 17.78% for non-COPD), “causative pathogens” (14.65% for COPD; 17.07% for non-COPD), and “not causative pathogens” (16.13% for COPD; 12.26% for non-COPD). In addition, causative pathogens were identified in the majority of samples from both subgroups (85.33% for COPD; 83.99% for non-COPD) (**Supplementary Figure S2**).

For causative or possibly causative pathogens, monthly trends over a period of more than one year (January 2024 - January 2025) demonstrated that gram-negative bacteria (GNB) consistently accounted for the largest share of detections - ranging from 30.92% to 61.45% of cases - with peaks in late spring (June) and autumn (November) (**Figure 3B**). Gram-positive bacteria (GPB) remained relatively stable at 10.29% - 34.30%. fungus comprised 5.49% - 15.52% of identifications and showed a gradual upward trajectory toward winter. Virus detections (both DNA and RNA viruses) exhibited clear seasonal oscillations, with RNA virus frequency rising 6.30% in November 2024 - January 2025. Atypical pathogens remained rare (2.69% - 14.71%) throughout.

When restricted to causative or possibly causative pathogens, GNB dominated both groups but significantly more so in COPD than in non-COPD patients (55.91% versus 45.53%) (**Figures 3C, D**). Furthermore, the prevalence of fungal infections was higher among patients with COPD compared to those without COPD (11.02% *vs*. 9.98%). In contrast, infections caused by gram-positive bacteria (21.69% *vs*. 19.09%), DNA viruses (8.19% *vs*. 5.12%), RNA viruses (7.33% *vs*. 6.89%), and atypical pathogens (7.27% *vs*. 1.97%) were more frequently observed in non-COPD patients.

At the species level, *Streptococcus pneumoniae* (21.62% for COPD; 18.60% for non-COPD) and *Klebsiella pneumoniae* (16.22% for COPD; 18.15% for non-COPD) were the two most frequently detected pathogens in both cohorts (**Figure 3E**). In COPD patients, *Pseudomonas aeruginosa* (21.62% versus 10.15%) and *Haemophilus influenzae* (15.44% versus 9.02%) were relatively enriched, whereas non-COPD patients exhibited higher detection rates of atypical (12.63% versus 2.70%) and viral (14.43% versus 10.04% for DNA virus; 12.40% versus 11.58% for RNA virus) pathogens such as *Mycoplasma pneumoniae* (11.39% versus 2.32%) and Human gammaherpesvirus 4 (7.89% versus 6.18%). Through a more detailed analysis, the extensive inter-patient heterogeneity was further proved. The heatmap in **Supplementary Figure S3** further highlighted extensive inter-patient heterogeneity: *Streptococcus pneumoniae* and *Klebsiella pneumoniae* maintained high occurrence across samples, while fungal and viral detections were more sporadic (distribution of all microorganisms was shown in **Supplementary Figure S4**).

The nucleic acid analysis against the causative or possibly causative pathogens showed no significant differences between COPD and non-COPD groups for total pathogens (p value = 0.8922), GPB (p value = 0.3514), GNB (p value = 0.0578), fungi (p value = 0.1056), DNA viruses (p value = 0.0843), or RNA viruses (p value = 0.9342) (**Figure 3F**). By contrast, atypical pathogens exhibited a significantly higher read count in non-COPD patients (p value = 0.0009).

In 259 COPD patients with respiratory infections, bacterial pathogens were the most frequently detected etiology, accounting for 71.43% (185/259) of samples (**Supplementary Figure S5A**). Pathogen-negative specimens comprised 14.67% (38/259), fungal infections 5.79% (15/259), viral infections 4.63% (12/259), atypical infections 1.16% (3/259), and mixed infections 2.32% (6/259). Among the six mixed infection cases, dual bacterial-viral co-infections predominated (66.67%, 4/6), while viral-atypical and triple bacterial-fungal-viral combinations each occurred in one patient (16.67%, 1/6, each). Human gammaherpesvirus 4 (n = 3) and *Streptococcus pneumoniae* (n = 2) were the most two common co-pathogens (**Supplementary Figure S5B**).

In 887 non-COPD patients, bacterial infection remained the dominant infection type (56.03%, 497/887), followed by atypical infections 10.03% (89/887) and viral infections 8.34% (74/887) (**Supplementary Figure S5C**). Mixed infections accounted for 4.74% (42/887) of samples and were predominantly bacterial-fungal (35.71%, 15/42), bacterial-viral (26.19%, 11/42), and bacterial-fungal-viral (11.90%, 5/42) combinations. Common co-pathogens included *Aspergillus fumigatus* (n = 13), *Streptococcus pneumoniae* (n = 11), and *Pneumocystis jirovecii* (n = 11) (**Supplementary Figure S5D**).

### 3.4 Characterization of non-pathogenic microorganisms

**Figure 4:**
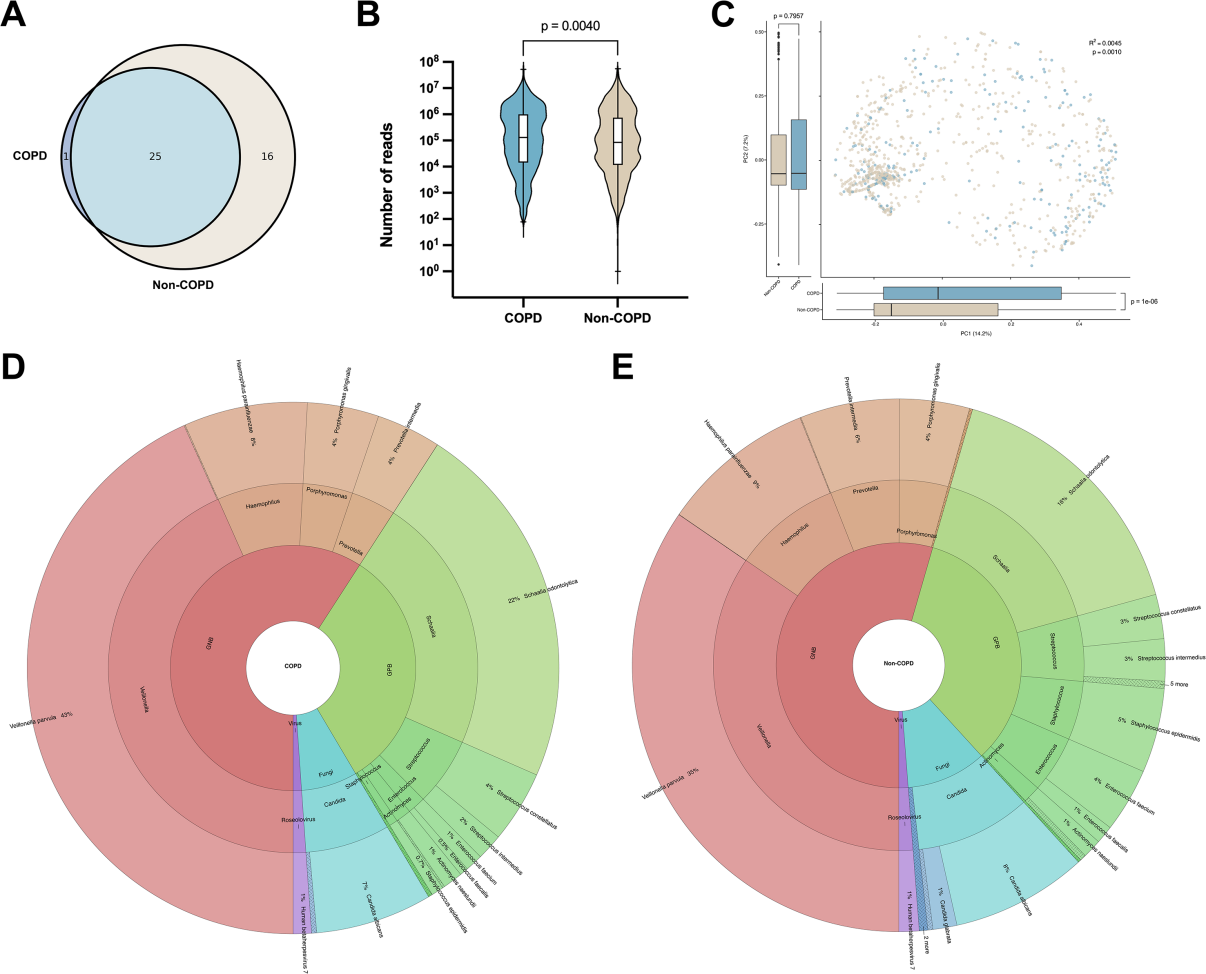
Distribution of non-pathogenic microorganisms. **(A)** Number of species of microorganisms identified in COPD versus non-COPD groups. The intersection is the number of species identified in both groups. **(B)** Distribution of reads counts of microorganisms detected by NGS in COPD versus non-COPD groups. **(C)** Principal coordinate analysis based on all non-pathogenic microbial detection information in COPD and non-COPD groups. PC1, principal coordinate one, the largest source of variance; PC2, principal coordinate two, the second largest source of variance. Percentages in parentheses are the degree of explanation of variance for the corresponding principal coordinate. **(D, E)** Distribution of microbial species and the types they belong to in COPD and non-COPD groups. GPB, gram-positive bacteria; GNB, gram-negative bacteria.

A total of 42 non-pathogenic microbial species were detected across both cohorts: only one species was unique to COPD group, 16 species were unique to non-COPD group, and 25 species were shared by both (**Figure 4A**). Loads of these microorganisms were significantly higher in COPD patients (median reads count 1.31×10^5^) than in non-COPD patients (median reads count 8.55×10^4^; p value = 0.004) (**Figure 4B**).

Principal coordinate analysis based on all non-pathogenic microbial detection data revealed clear separation along the first axis: PC1 accounted for 14.2% of the variance and differed markedly between COPD and non-COPD groups (p value < 0.0001), whereas PC2 (7.2% of variance) showed no significant difference (p value = 0.7957). PERMANOVA confirmed that group membership explained a small but statistically significant fraction of the overall variance (R^2^ = 0.0045, p value = 0.001) (**Figure 4C**).

The overall distribution of microbial species was comparable between COPD and non-COPD groups, with a predominance of GNB observed in both cohorts (59% for COPD; 54% for non-COPD), followed by GPB (32% for COPD; 34% for non-COPD), fungi (7% for COPD; 11% for non-COPD), and viruses (1% in both groups) (**Figures 4D, E**). However, species-level differences were noted between the groups. *Veillonella parvula* and *Schaalia odontolytica*, the predominant microorganisms, accounted for a higher proportion of isolates in COPD patients (43% and 22%, respectively) compared to non-COPD patients (35% and 16%, respectively). In contrast, several GPB species were more frequently detected in non-COPD group, including *Staphylococcus epidermidis* (5% versus 0.7%), *Enterococcus faecium* (4% versus 1%), *Streptococcus intermedius* (3% versus 2%), and *Enterococcus faecalis* (1% versus 0.5%).

### 3.5 Machine learning model predicts poor prognosis

**Figure 5:**
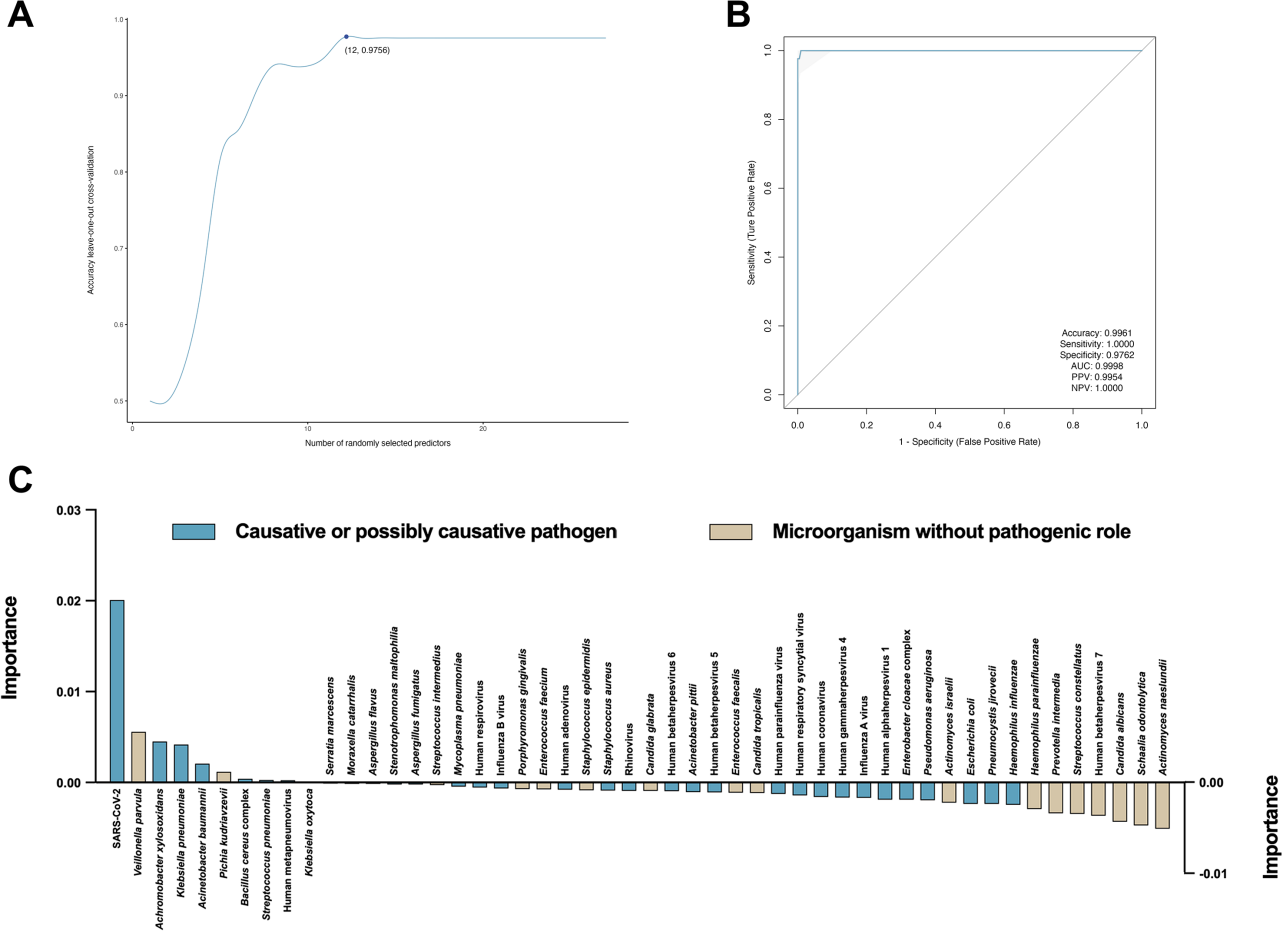
Machine learning for predicting poor prognosis of respiratory infections in COPD patients. **(A)** Cross-validation of hyperparameter screening for the random forest model. **(B)** Performance of the random forest model in bootstrap sampling-based test set. AUC, area under the receiver operating characteristic curve; PPV, positive predictive value; NPV, negative predictive value. **(C)** Importance of factors extracted from the random forest model.

To delineate the specific microbial features driving clinical outcomes, we employed a random forest (RF) machine learning approach coupled with rigorous feature selection protocols (**Supplementary Figure S6**). We utilized recursive feature elimination (RFE) integrated with leave-one-out cross-validation (LOOCV) to objectively determine the optimal subset of predictive markers. This process iteratively evaluated model performance while systematically pruning the least informative features. As illustrated in **Figure 5A**, the model’s predictive accuracy improved rapidly with the inclusion of initial high-value features and achieved a performance plateau at a parsimonious set of 12 microbial predictors. The inclusion of additional features beyond this point did not significantly enhance discriminative power, suggesting that 12 markers capture the maximal prognostic signal while minimizing the risk of overfitting. Using this optimized hyperparameter, we assessed model performance on a bootstrap-resampled test set to ensure robustness. The classifier demonstrated exceptional discriminative ability, yielding an area under the receiver operating characteristic curve (AUC) of 0.9998 (**Figure 5B**). The model achieved a sensitivity of 100.00%, specificity of 97.62%, positive predictive value (PPV) of 99.54%, and negative predictive value (NPV) of 100.00%, with an overall accuracy of 99.61%. These metrics highlight the signature’s reliability in distinguishing between patients with poor versus favorable clinical trajectories.

To clarify the contribution of individual microbes, we analyzed feature importance using the mean decrease in Gini index (**Figure 5C**). This analysis identified SARS-CoV-2 abundance as the dominant predictor of poor prognosis (weight = 0.0201), significantly outranking other pathogens. Following SARS-CoV-2, the hierarchy of risk-associated features included *Veillonella parvula* (weight = 0.0056), *Achromobacter xylosoxidans* (weight = 0.0045), and *Klebsiella pneumoniae* (weight = 0.0042). Conversely, directional analysis of the feature contributions revealed distinct protective profiles. Specifically, *Actinomyces naeslundii* (weight = -0.0051), *Schaalia odontolytica* (weight = -0.0047), and *Candida albicans* (weight = -0.0043) exhibited inverse associations with the primary outcome, suggesting a potential protective role or their depletion in high-risk phenotypes. These confirm that high viral loads (SARS-CoV-2) and elevated specific bacterial pathogens serve as the primary nodes for stratifying high-risk patients, underscoring a complex, non-linear hierarchical interaction between viral drivers and the bacterial microbiome in determining disease progression.

## 4 Discussion

In this comprehensive investigation, we characterized the microbial profiles in COPD patients compared with non-COPD individuals, demonstrating notable differences in pathogenic and non-pathogenic microorganisms and their correlation with clinical outcomes. Utilizing NGS, we highlighted distinct microbiological landscapes and identified key pathogens significantly associated with adverse clinical outcomes in COPD patients.

Consistent with prior studies, bacterial pathogens predominated in respiratory infections among both COPD and non-COPD groups (Ieven et al., 2018; Li et al., 2021; Shmoury et al., 2023). Importantly, our findings demonstrate a marked enrichment of GNB, particularly *Pseudomonas aeruginosa* and *Haemophilus influenzae*, among COPD patients. These pathogens have previously been implicated in recurrent exacerbations, rapid disease progression, and impaired lung function, potentially due to chronic colonization facilitated by altered airway immunity and compromised mucociliary clearance characteristic of COPD (McDonnell et al., 2015; Araújo et al., 2018; Eklöf et al., 2020; Lindgren et al., 2021).

Interestingly, the incidence of fungal infections was higher among COPD patients. This aligns with earlier evidence suggesting increased susceptibility of COPD patients to fungal colonization and infection, possibly attributable to prolonged corticosteroid therapy, frequent antibiotic exposure, and impaired local immunity (Tiew et al., 2020; Waqas et al., 2020; Wu et al., 2021; Tiew et al., 2022). The presence of fungi such as *Aspergillus* has previously been associated with worse clinical outcomes, indicating the necessity for clinicians to maintain heightened vigilance for fungal pathogens in managing COPD exacerbations.

In contrast, atypical pathogens, including *Mycoplasma pneumoniae*, were significantly more prevalent in non-COPD patients. This discrepancy could stem from differences in host immunity or microbiome composition, warranting further exploration into the immunopathological mechanisms underpinning susceptibility to atypical pathogens in the non-COPD population (Narita, 2016; Fan et al., 2024; Georgakopoulou et al., 2024). Additionally, the seasonal trends observed in viral infections underscore the importance of preventive strategies, including targeted vaccination programs, to mitigate COPD exacerbation risks during peak viral transmission periods.

Another critical finding of our study is the enrichment of microorganisms traditionally classified as non-pathogenic, particularly *Veillonella parvula* and *Schaalia odontolytica*, in patients with COPD. Emerging evidence from microbiome-immune interaction research suggests that such commensal taxa can exert context-dependent immunomodulatory effects rather than remaining immunologically inert. *Veillonella* species, for instance, have been shown to influence local immune tone through the production of short-chain fatty acids and other metabolites capable of shaping neutrophil recruitment, Th17 polarization, and mucosal barrier integrity (Gu et al., 2020; Hays et al., 2024). In the chronically inflamed airway milieu of COPD, these mechanisms may promote a state of dysregulated immune tolerance, amplifying low-grade inflammation and impairing effective pathogen clearance. Moreover, non-pathogenic microorganisms may participate in cooperative or competitive interactions within microbial communities, indirectly enhancing the virulence or persistence of established pathogens through metabolic cross-feeding or biofilm stabilization (Ramsey et al., 2011; Wang et al., 2023). Such microbiome-driven immune reprogramming provides a plausible biological framework linking the observed microbial shifts to disease severity and exacerbation susceptibility. Future longitudinal studies integrating functional metagenomics, metabolomics, and host immune profiling will be essential to delineate the causal pathways through which these organisms contribute to COPD pathogenesis and to determine whether they represent viable targets for microbiome-based therapeutic interventions.

Our study leverages a supervised machine learning framework to move beyond simple abundance comparisons, enabling the identification of microbial features predictive of clinical deterioration. Crucially, the feature importance analysis reaffirms the central role of SARS-CoV-2 burden as the primary driver of poor outcomes, a finding that aligns with established pathophysiology but is here quantified relative to co-infecting agents (Bowe et al., 2022; Lee et al., 2025). Beyond the viral trigger, the model successfully disentangled the roles of secondary bacterial actors. The identification of *Achromobacter xylosoxidans* and *Klebsiella pneumoniae* as key predictive features supports the hypothesis that secondary bacterial superinfections or dysbiosis significantly exacerbate disease severity in vulnerable COPD populations (Marsac et al., 2021; Ouyang et al., 2024). Furthermore, the model’s identification of “protective” commensals (e.g., *Actinomyces naeslundii*) provides a nuanced view of the respiratory ecosystem, suggesting that the loss of colonization resistance may be as critical as pathogen acquisition. By integrating these hierarchical signals, our machine learning approach provides a roadmap for developing targeted pathogen-specific therapeutic strategies and enhanced monitoring protocols, potentially enabling earlier intervention before progression to severe clinical endpoints.

Our study has several notable strengths, including a large cohort size, utilization of highly sensitive and specific NGS technology, and comprehensive analysis encompassing both pathogenic and traditionally non-pathogenic microorganisms. Nonetheless, certain limitations should be acknowledged. Firstly, our study was conducted at a single institution using a single sample type, BALF, potentially limiting generalizability. Secondly, the observational design precludes establishing causal relationships between microbial profiles and clinical outcomes. Prospective, multicenter studies with extended follow-up periods are warranted to validate our findings and further elucidate the mechanistic pathways linking microbial communities to COPD exacerbations and prognosis. Finally, because the primary objective of this study was biomarker identification, model optimization for generalizability was not prioritized. Consequently, potential overfitting was not addressed, which may limit the applicability of the model’s feature weights to external cohorts.

In conclusion, our findings provide critical insights into the microbiological underpinnings of COPD-related infections, highlighting pathogen-specific associations with clinical prognosis. These results have significant implications for clinical practice, suggesting that precise microbial identification via NGS could facilitate personalized therapeutic strategies, ultimately improving patient outcomes in COPD.

## Data Availability

The original contributions presented in the study are included in the article/**Supplementary Material**. Further inquiries can be directed to the corresponding author.
