# Supplementary material for: Characteristics of pathogenic microorganisms in COPD-related infections: prognostic correlations and implications
Source: Front Cell Infect Microbiol. 2026 Jan 19;15:1739849. doi: 10.3389/fcimb.2025.1739849 (PMC12862076; doi:10.3389/fcimb.2025.1739849)
Supplement: Supplementary file 1 [file SupplementaryFile1.docx]

Supplementary Material

# Supplementary Figures and Tables

## Supplementary Figures


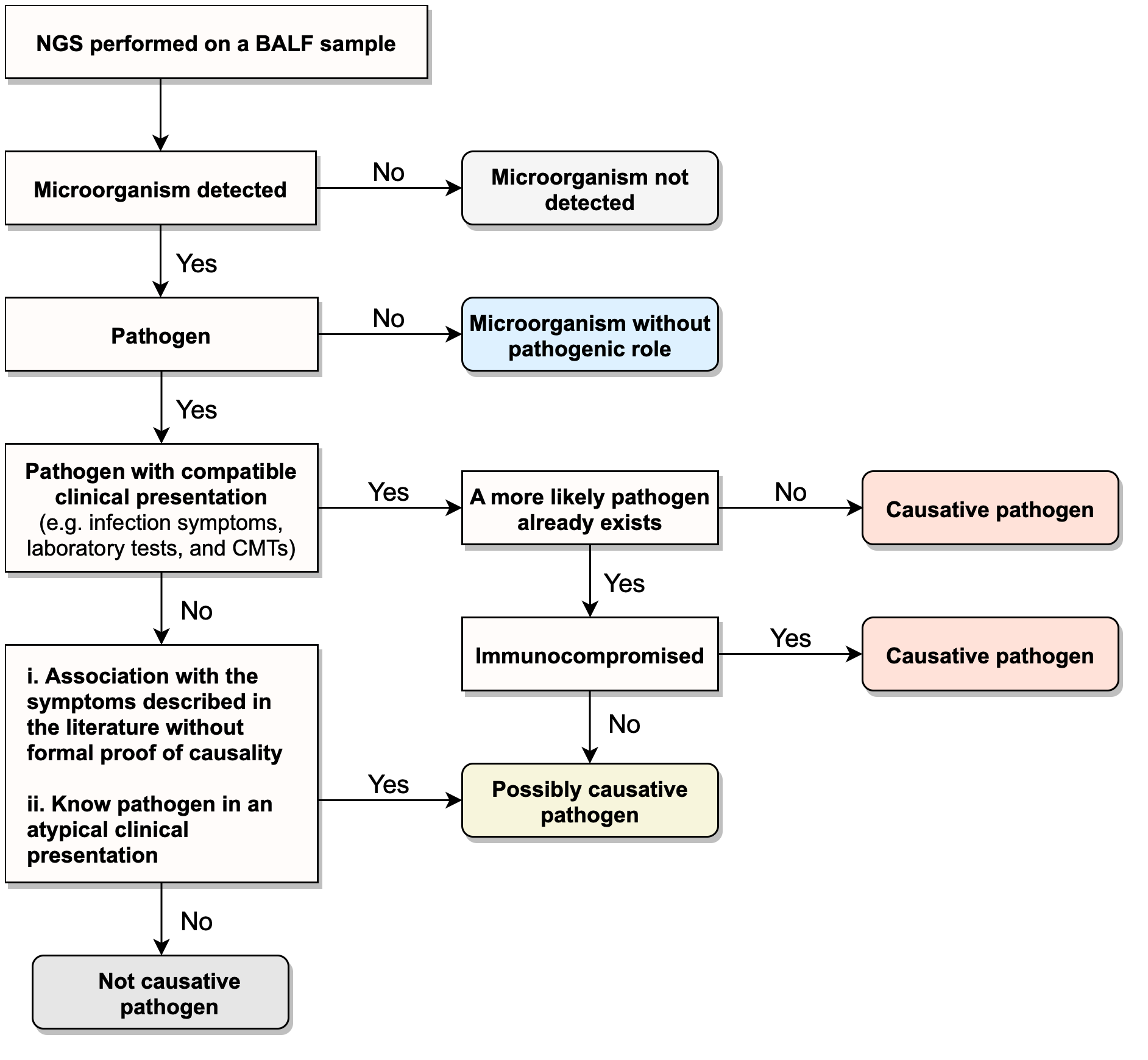


**Supplementary Figure 1.** Workflow of clinical diagnosis. NGS, next-generation sequencing; BALF, bronchoalveolar lavage fluid; CMT, conventional microbiological test.

## Supplementary Figures


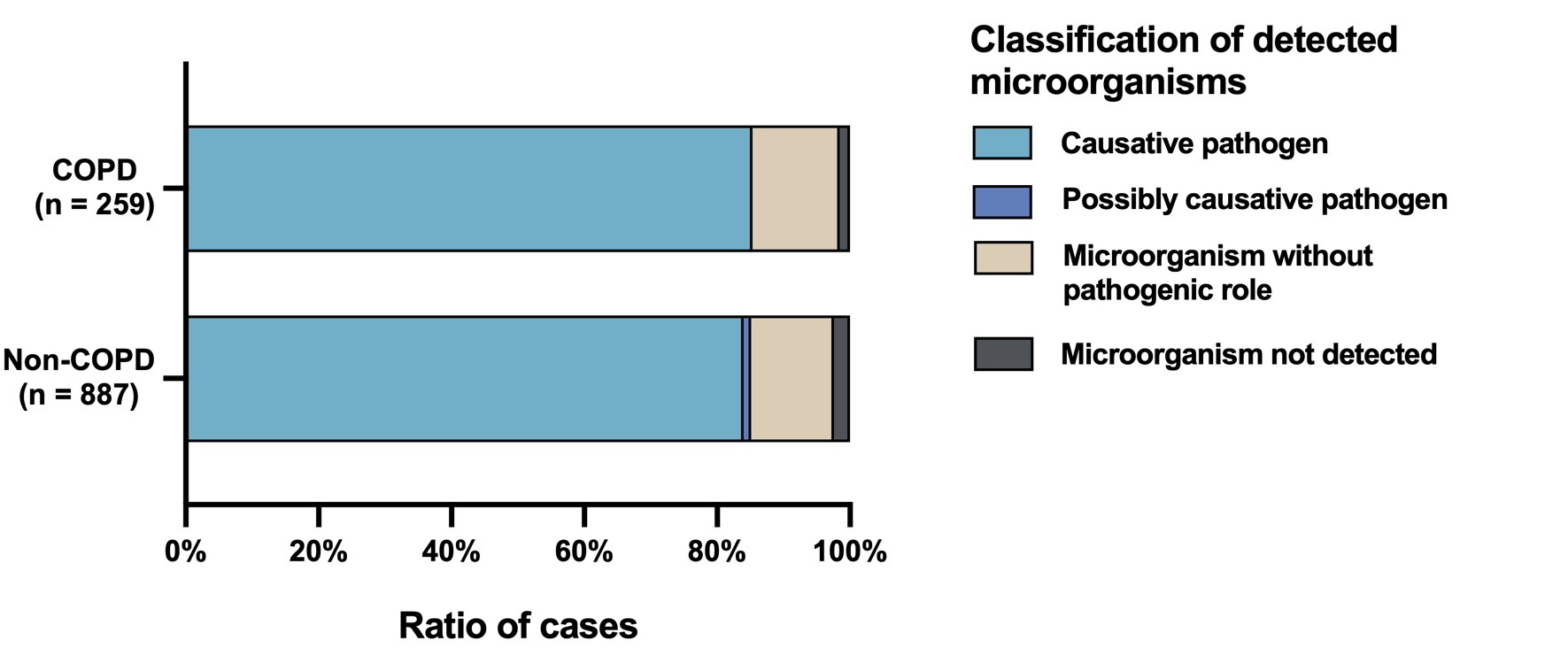


**Supplementary Figure 2.** All samples from patients with and without COPD, along with the proportional distribution across each corresponding level of clinical diagnosis. COPD, chronic obstructive pulmonary disease.

## Supplementary Figures


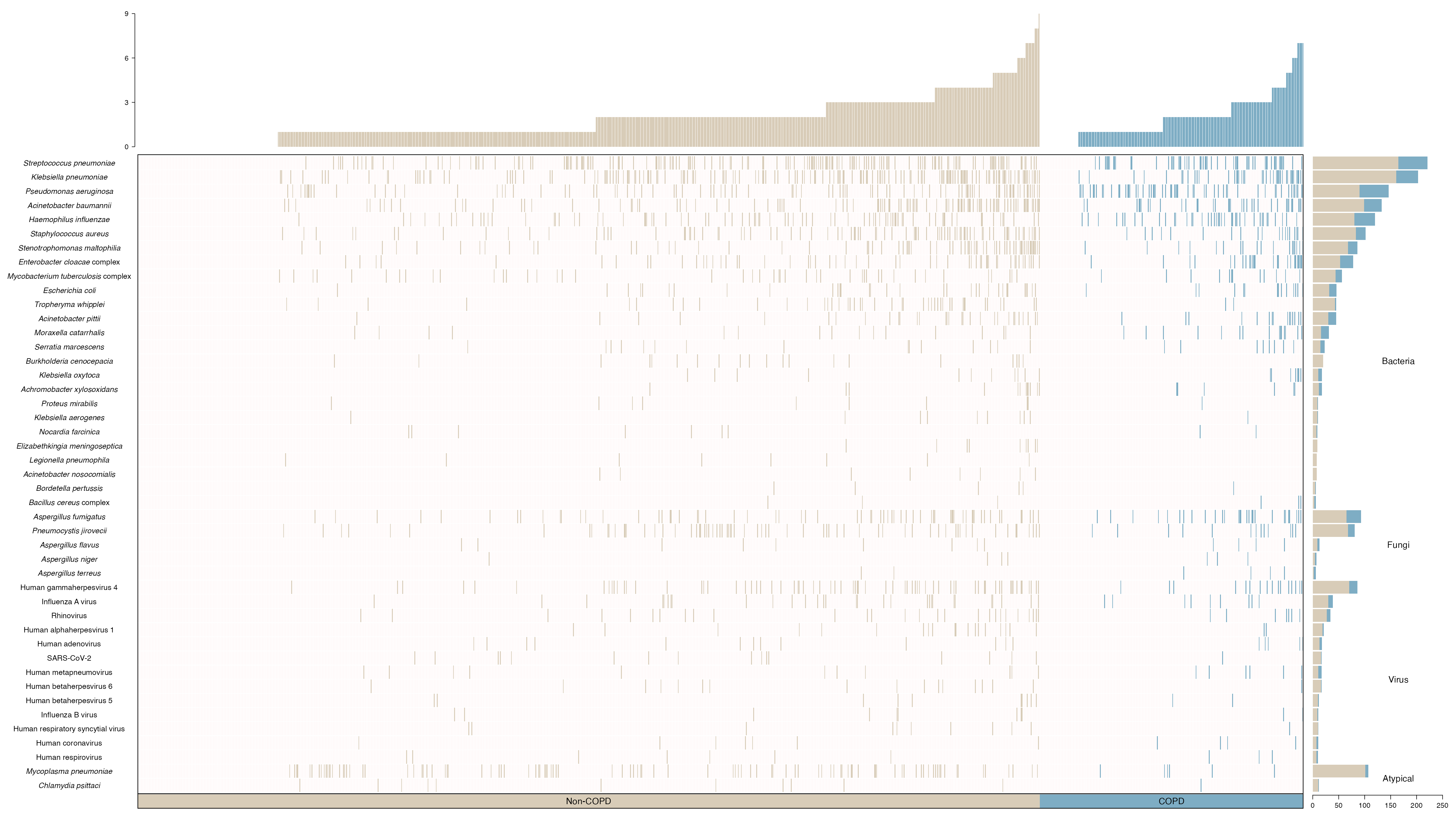


**Supplementary Figure 3.** Distribution of each species of causative or possibly causative pathogens detected in all samples. Bars are the frequency statistics.

## Supplementary Figures


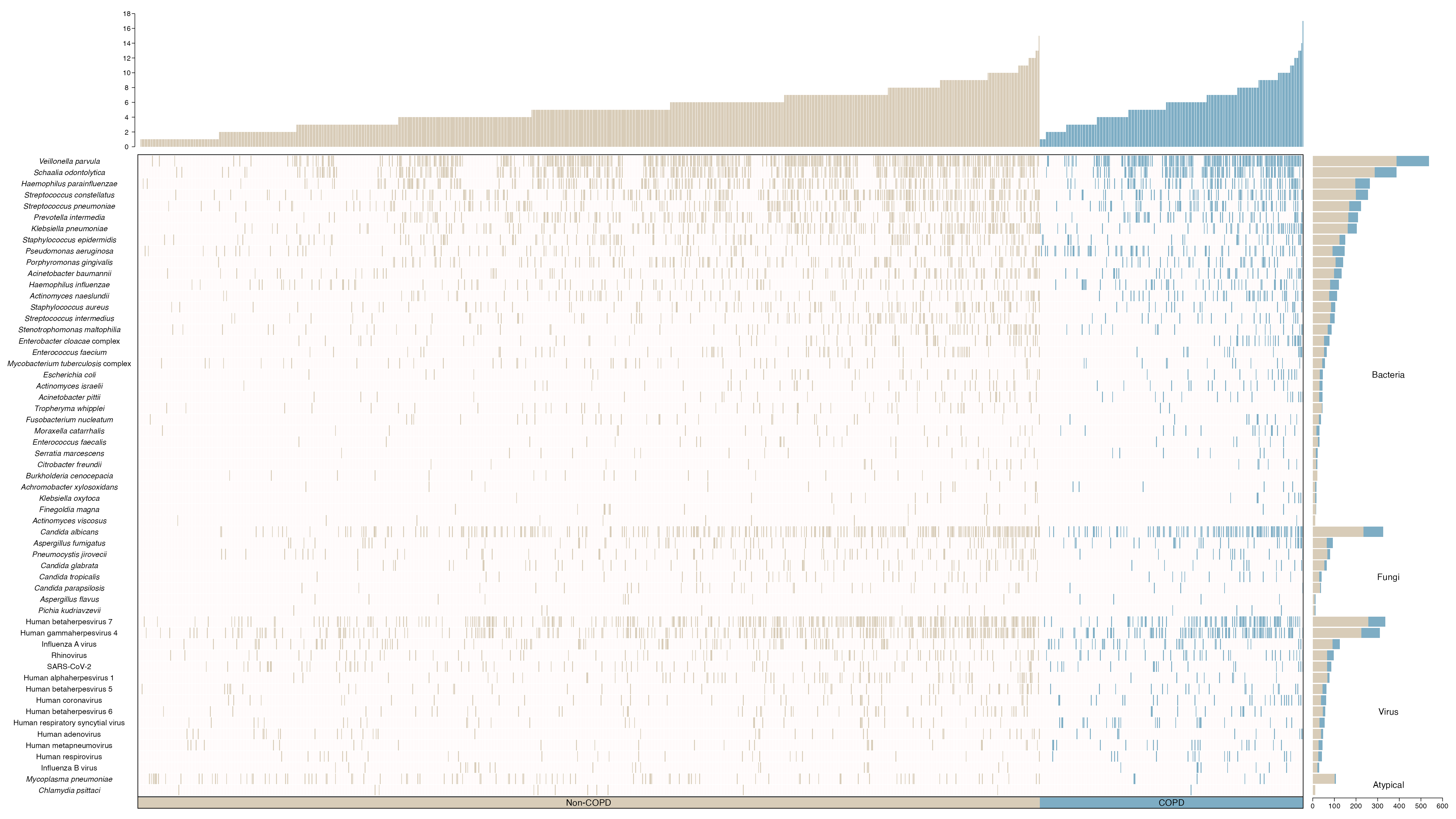


**Supplementary Figure 4.** Distribution of each species of microorganisms detected in all samples. Bars were the frequency statistics. COPD, chronic obstructive pulmonary disease.

## Supplementary Figures


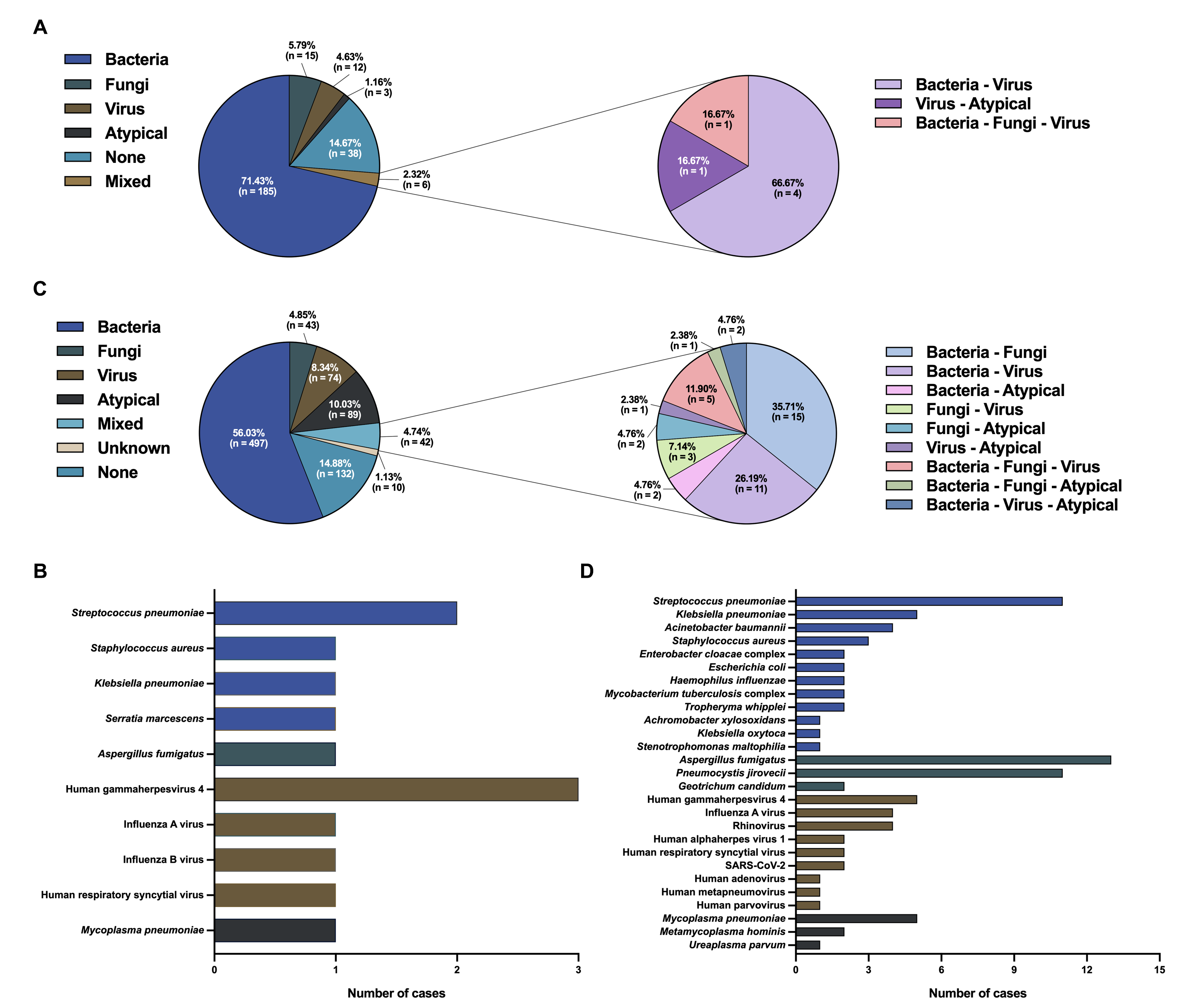


**Supplementary Figure 5.** Analysis of infection types in COPD and non-COPD patients. (A) Distribution of infection types in COPD patients. The left pie represents the types of causative or possibly causative pathogen detected in all samples from COPD patients. The right pie represents the types of mixed infection. (B) Frequency of causative or possibly causative pathogens in mixed infections of COPD patients. (C) Distribution of infection types in non-COPD patients. The left pie represents the types of causative or possibly causative pathogen detected in all samples from non-COPD patients. The right pie represents the types of mixed infection. (D) Frequency of causative or possibly causative pathogens in mixed infections of non-COPD patients.

## Supplementary Figures


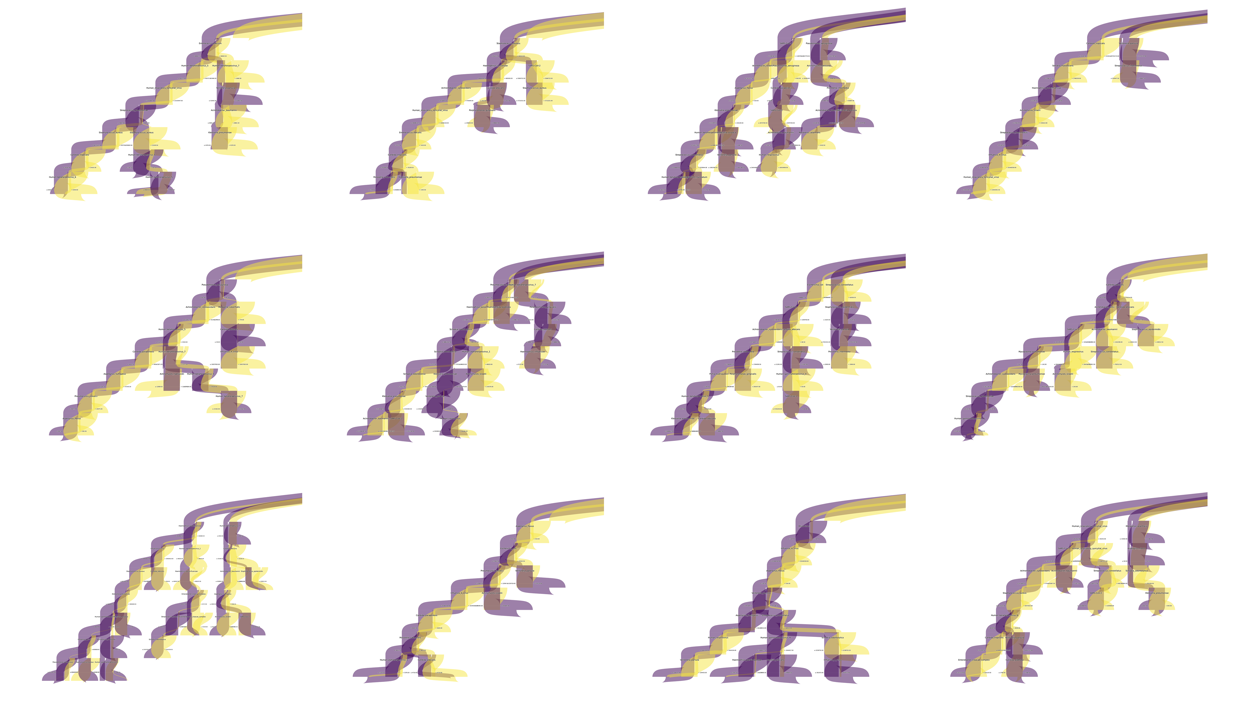


**Supplementary Figure 6.** Partial decision trees in the random forest model for predicting poor prognosis of respiratory infections in COPD patients.
